# 

**Published:** 1872-04

**Authors:** 


### BOOK NOTICES.

"Baldwin's Monthly" (edited and published by Baldwin the Clothier) is one of the prompt and welcome visitors to our sanctum. The March number comes to us with interesting original contributions, from several authors of note. The editorial columns bristle with sharp, pointed paragraphs relating to trade, which are in marked contrast to the hackneyed style of introducing a business to the public. The article which appears in the first place among the original contributions, is by Laura C. Holloway, and is written in the author's best vein, entitled "Memories of Actors and Actresses." The selections from other journals evince a painstaking seldom seen in the monthlies of the present day.

Baldwin's is the largest ready-made clothing business in the United States. His sales at *retail*, amount to over twelve hundred thousand dollars annually.

We are authorized to say that Baldwin the Clothier, corner Canal and Broadway, New York, will place any name on his list and send the Monthly free, if the address is sent to him. Each number will be embellished with an original engraving.

"Insanity, Plain Talk about it; its Causes, Forms, Symptoms, and the Treatment of Mental Diseases; with Remarks on Hospitals and Asylums, and the Medico-Legal Aspect of Insanity," by T. W. Fisher, M. D., late of the Boston Hospital for the Insane. $1.50. Published by Alexander Moore, Hamilton Place, Boston, Mass. One of the most suggestive books yet published on the subject.

"The Aldine," is a monthly pictorial periodical, the most elegant and artistical in this country. The editor is Richard H. Stoddard, an able writer and a ripe scholar; the business director is Mr. Sutton, a man of indomitable energy and enterprise. Under such auspices, a grand success will doubtless be achieved.

